# The Emission Reduction Effect of Financial Agglomeration under China’s Carbon Peak and Neutrality Goals

**DOI:** 10.3390/ijerph20020950

**Published:** 2023-01-04

**Authors:** Yanan Wu, Biyu Peng, Yehui Lao

**Affiliations:** 1 School of Economics & Management, South China Normal University, Guangzhou 510006, China; 2 College of Economy and Trade, Zhongkai University of Agriculture and Engineering, Guangzhou 510225, China

**Keywords:** financial agglomeration, emission reduction effect, carbon peaking and carbon neutralization, spatial effect, carbon emissions trading

## Abstract

The existing literature on the influencing factors of carbon emissions ignores the relationship between financial agglomeration and carbon emissions. Based on the analysis of the emission reduction history of major countries, this paper mainly uses the provincial-level data of China from 2002 to 2018 to explore the impact of financial agglomeration on carbon emissions. The conclusions are as follows: (1) China lacks carbon tax policies; there are many drawbacks in the carbon trading market, and a “bottom-up” voluntary emission reduction mechanism has not been formed. (2) China’s carbon emissions and financial development are characterized by spatial agglomeration. (3) Financial agglomeration can reduce carbon emissions. In central China, the low-carbon region, and the pilot regions for carbon trading, financial agglomeration has a greater impact on reducing emissions. (4) Financial agglomeration can reduce emissions by reducing the proportion of the secondary industry and increasing the proportion of the third industry. (5) Financial agglomeration can still lower carbon emissions when the spacing effect is taken into account. Finally, according to the conclusion, this paper puts forward relevant suggestions to help China reduce carbon emissions.

## 1. Introduction

Excessive greenhouse gas emissions will lead to the superposition of various extreme climate crises, posing a serious threat to humans, economic development, food security, biodiversity, and ecosystems [1,2]. If global carbon emissions increase at the current rate, the global temperature may increase by 1.5 °C from 2030 to 2052 and even exceed 3–5 °C by the end of the 21st century [3]. Reducing carbon emissions is an important strategy for dealing with the climate crisis [4]. Various countries have also launched actions to address climate change. At present, major developed countries such as the European Union, the United States, Germany, the United Kingdom, and Japan have reached carbon peaks one after another. They have committed to carbon neutralization by 2050 in the form of political agreements, strategies, laws, and statements of intent. In 2019, China’s total carbon emissions were about four times that of the EU, twice that of the United States, and 16 times that of Germany. China’s total carbon emissions are rising and have not yet reached carbon peaks. However, China promises to achieve peak carbon emissions by 2030 and carbon neutrality by 2060. The accompanying pressure on emission reduction is greater. Therefore, China urgently needs to adopt comprehensive policies to reduce emissions.

The Solow model says that, in a perfectly competitive market, economic growth is stable, and the market can reach Pareto optimality [5]. Most scholars believe that, when private interests and social interests are inconsistent, the market cannot achieve the Pareto optimal state, and then externalities occur [6]. Excessive carbon emissions from human activities are a typical example of a negative external economy. These emissions threaten the safety of people and their property. Governments and other institutions must skillfully intervene in the market and formulate effective environmental policies to govern negative economic externalities [7,8]. Pigou proposed that measures such as taxation, formulating emission standards, levying pollution charges, and issuing pollution permits can be taken to internalize external costs to control carbon emissions [9]. Baumol and Oates proposed establishing environmental quality standards to govern negative externalities [10]. Marshall believes that there is an external economic effect between industries, that is, the supporting effect of one industry on another [11]. Therefore, the external economic effects between industries can be used to control carbon emissions. Government agencies and academia have noted that the financial industry can impact on carbon emissions.

Financial support is required to achieve the two goals of “carbon peak” and “carbon neutrality” as soon as possible. Therefore, in February 2021, the State Council of China issued the Guiding Opinions on Accelerating the Establishment and Improvement of a Green Low-Carbon Circular Development Economic System, which suggested developing green credit, green direct financing, green insurance, and climate investment and financing. In March 2021, the People’s Bank of China proposed to give play to the functions of financial resource allocation, risk management, and market pricing. They will contribute to carbon peak and carbon neutrality by improving the green financial standard system, strengthening supervision and information disclosure, improving incentive and restraint mechanisms, enriching green financial products, and expanding the international green finance cooperation space. At the same time, the Ministry of Ecology and Environment of China issued the Interim Regulations on the Administration of Carbon Emission Trading (Revised Draft), proposing to establish a national carbon emission trading fund and attaching importance to the financial attributes of the carbon market. Therefore, finance will play an important role in the process of carbon emission reduction in China.

Some scholars believe that finance has increased carbon emissions by promoting production, increasing energy consumption, and promoting commodity sales [12,13,14]. Some scholars believe that finance can reduce carbon emissions by promoting companies’ energy-saving technologies, controlling environmental pollution, improving environmental quality, and improving resource utilization efficiency [15,16]. Some scholars believe there is a nonlinear relationship between finance and regional carbon emissions [17]. Previous studies expanded and enriched the scope of financial development affecting carbon emissions but ignored the impact of financial spatial agglomeration characteristics on regional carbon emissions.

The phenomenon of various financial entities gathering in specific regions is called financial agglomeration. Analyzing the relationship between the spatial agglomeration of economic variables and economic phenomena belongs to the new economic geography, which was created by Paul Krugman [18]. After the development of Venables [19], Baldwin [20], Martin [21], and others, this theory has become a significant advantage in analyzing the spatial characteristics and economic significance of economic activities. The existing literature has also studied the role of financial agglomeration. However, few works of literature have studied the impact of financial agglomeration on regional carbon emissions.

To make up for the shortage of existing literature, we designed the following research: First, this paper analyzes the research background, including the carbon emission status and related policies of major countries. Then, we use econometric models to analyze the direct impact, impact mechanism, heterogeneity analysis, and spatial effect of financial agglomeration on carbon emissions. Finally, we put forward relevant suggestions according to the finding. The research design of this paper makes up for the lack of existing research and has theoretical and practical significance.

The rest is organized as follows: The section on “Research background and hypotheses” reveals the carbon emission status and emission reduction policies of China and other major countries worldwide and introduces research hypotheses. The section on “Materials and methods” shows the research design of this paper. The “Imperial Results and Analysis” section reviews the impact, impact mechanism, and spatial effect of financial agglomeration on regional carbon emissions. Finally, the “Conclusion and Suggestions” part summarizes the research and provides relevant suggestions.

## 2. Research Background and Hypotheses

### 2.1. The Current Level of Carbon Emissions

**Figure 1:**
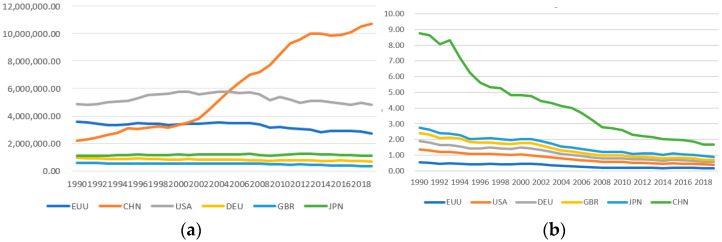
(**a**) Change trend of total carbon emissions; (**b**) change trend of carbon emission intensity. Data source: World Bank.

**Figure 2:**
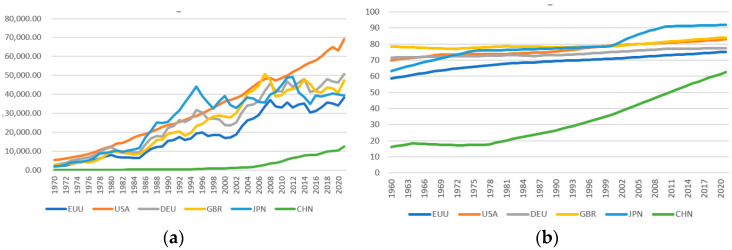

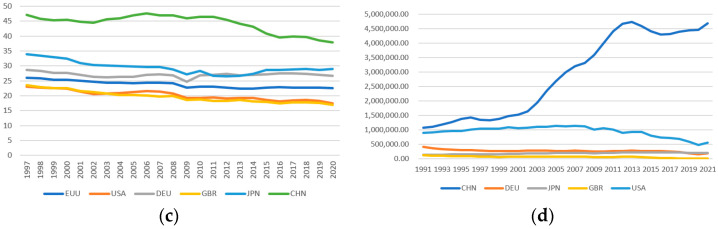
(**a**) GDP per capita (current USD); (**b**) urbanization rate; (**c**) proportion of secondary industry; (**d**) coal consumption (MST); data source: National accounts data files of the World Bank and the OECD; United Nations, World Urbanization Prospects; U.S. Energy Information Administration.

Figure 1a shows that China’s total carbon emissions are rising. China’s carbon emissions growth rate was the fastest from 2000 to 2014, and the growth rate was relatively slow from 2015 to 2019. In 2019, China’s total carbon emissions will be about four times that of the EU, twice that of the United States, and 16 times that of Germany. Germany, Britain, and Japan have the lowest total carbon emissions. The total carbon emissions of the European Union have been declining since 1990 and the United States since 2007. Figure 1b shows that China’s carbon emissions per unit of GDP have been declining since 1993, but the carbon emission intensity is still the highest. The carbon emission intensity of other countries is also in a declining trend. Figure 2a–d shows that China’s per capita GDP is lower, that the urbanization rate is lower, that the proportion of the secondary industry is higher, and that the coal consumption is higher.

### 2.2. Major Country Emission Reduction Policies

#### 2.2.1. The EU’s Emissions-Reduction Experience

The European Union has always been an active advocate for the climate crisis. The EU put forward the 2020 Climate and Energy Package in 2007. The plan attempts to reduce emissions by 20 percent from 1990 levels. In 2011, the EU adopted the Energy Roadmap 2050 and the Roadmap towards a Competitive Low Carbon Economy 2050, which set a long-term goal of reducing greenhouse gas emissions by 80–95% by 2050. In 2014, the European Union proposed the 2030 Climate and Energy Policy Framework to reduce greenhouse gas emissions by 40% in 2030 based on 1990. The European Union adopted the European Energy Efficiency Designation in 2019 to improve energy efficiency by 32.5% by 2030. At the same time, the EU put forward the EU’s 2030 and 2050 climate goals in the European Green Agreement. The European Climate Law adopted in 2020 to ensure carbon neutrality by 2050 in legal form.

The major methods used by the EU to reduce carbon emissions are carbon taxes and emissions trading. The EU carbon emission trading system was officially launched in 2005 and has gone through four stages. The first stage was from 2005 to 2007. The EU’s emission reduction targets are mainly based on the Kyoto Protocol. The second phase is 2008–2012. Based on 2005, the EU set a target of 6.5% emission reduction. The third stage is from 2013 to 2020. The EU’s emission reduction goal is a 20% drop in emissions from 1990 levels. The fourth stage is 2021–2030. The EU will introduce the Market Stability Reserve (MSR) mechanism and strive to reduce emissions by 43% based on 2005 and 40% based on 1990 in 2030. From the perspective of the carbon tax, since 1990, the EU has gradually formed a carbon tax system that is separately disbursed; represented by Finland, Sweden, and Norway; and collectively paid by Italy and Germany. The entire carbon tax system in the EU is developed, with a wide range of taxation methods and a high rate of taxes. After 2018, the EU gradually implemented a linking policy between the carbon trading market and the carbon tax in order to put more financial pressure on high-emission businesses and strengthen the efficiency of the carbon tax system.

Germany, a significant member of the European Union, actively promotes the growth of electric transportation through its economic recovery plan, finances the development of new energy sources and green technologies, and supports the use of low- and zero-emission vehicles. The UK published Promoting a Zero Carbon Future in 2020 with the intention of reducing carbon emissions through the robust development of wind power, supporting CCUS cluster construction, developing digital infrastructure construction, low-carbon industrial clusters, and a green economy.

#### 2.2.2. The US’s Emissions-Reduction Experience

Since 1993, the United States has implemented several policies and laws, such as the National Environmental Policy Act, the Comprehensive Response, the 2007 Low Carbon Economy, and so on. The USA also adopted several strategies to improve energy efficiency, improve the energy structure, boost the economy, and address climate change, such as the National Energy Comprehensive Strategy, the Green Economy Recovery Plan, and the Administrative Order to Address Domestic and Foreign Climate Crises. At the same time, the US encourages businesses to create energy conservation and emission-reduction technologies, as well as carbon dioxide recovery and storage technology, through financial subsidies, environmental taxes, tax credits, tax incentives, financial subsidies, and other measures. Additionally, they have gradually formed financial means and market mechanisms to promote carbon emission reduction at the enterprise level and reduce carbon emission through a “bottom-up” voluntary emission reduction model. In particular, the United States promotes energy transformation and accelerates low-carbon development in all sectors by increasing investment in clean energy and optimizing market resource allocation through green finance.

#### 2.2.3. China’s Emission Reduction Policy

Since joining the United Nations Framework Convention on Climate Change in 1992, China has signed international agreements such as the Kyoto Protocol, the Copenhagen Agreement, and the Paris Agreement. In 2007, China established a national plan to address climate change. In 2010, it carried out pilot work for low-carbon cities in five provinces and eight cities and formally implemented the Environmental Protection Tax Law in 2018. From the perspective of carbon emissions trading, China started the pilot work of carbon emissions trading in 2011, started trading in the carbon market in 2013, and launched the national carbon emissions trading market in 2021. China’s national independent contribution to addressing climate change and reducing carbon emissions has been increasing. In 2020, China promised to achieve a carbon peak by 2030 and carbon neutrality by 2060.

China has formed a “1 + N” policy system; 1 refers to the Opinions on Fully, Accurately, and Comprehensively Implementing the New Development Concept and Doing a Good Job of Carbon Peak Carbon Neutralization. This opinion is the system planning and overall deployment of carbon peaking and carbon neutralization, covering two stages and playing a leading role in the relevant policy system. N refers to a series of policy documents led by the 2030 Carbon Peak Action Plan, covering implementation plans, support measures, and safeguard measures in the major areas, industries, and regions. Finance is one of these safety measures. China actively promotes green finance and works to use the financial sector to support efforts to reduce carbon emissions.

### 2.3. Research Hypotheses

Germany provides financial support for new energy R&D and green technology innovation. The United States promotes energy transformation by optimizing market resource allocation through green finance. China’s “1 + N” policy system encourages green finance to play a role in resource allocation, risk management, and market pricing. Therefore, finance plays an important role in emission reduction. A region with strong financial development is more capable of promoting energy structure transformation to reduce regional carbon emissions. Meanwhile, financial agglomeration can promote the development of low-energy and green industries, improve energy consumption efficiency, and reduce carbon emissions. Based on this, this paper proposes hypothesis H0:

**H0.**  *Financial agglomeration can reduce regional carbon emissions*.

Secondary industrial carbon dioxide emissions have always accounted for a significant share of total carbon emissions. Financial agglomeration promotes enterprise competition and urges the high-carbon secondary industries to transfer to other regions due to high competition, high environmental-pollution control costs, and strict government regulation. The third industries generate fewer carbon emissions, have high added value, and easily survive fierce market competition. Hence, third industries can utilize financial resources more effectively. Based on this, this paper proposes hypotheses H1 and H2:

**H1.**  *Financial agglomeration can reduce carbon emissions by reducing the proportion of secondary industry*.

**H2.**  *Financial agglomeration can reduce carbon emissions by increasing the proportion of third industry*.

The European Union, the United States, China, and other countries adopted carbon trading to control carbon emissions. The government allocates tradable carbon emission quotas to emission control institutions. The quota can be bought and sold on the trading market. Enterprises with high-carbon emissions will buy carbon emission quotas from the market. Carbon emission trading increases the production costs of high-carbon enterprises, forcing them to reduce carbon emissions. Financial agglomeration and the carbon market can complement each other in the fields of expertise, information, institutions, and services. Financial agglomeration can provide financing tools for the carbon market, which helps to play up the financial attributes of the carbon market. Based on this, this paper proposes hypothesis H3:

**H3.**  *Carbon emission trading can strengthen the emission reduction effect of financial agglomeration*.

## 3. Materials and Methods

### 3.1. Samples and Data

Due to the serious lack of data from China’s Tibet Autonomous Region, Hong Kong, Macao, and Taiwan, this paper focuses on the relevant data of 30 provinces, autonomous regions, and municipalities in China from 2002 to 2018. The variables are from the Guotai’an Database, the China Statistical Yearbook, provincial statistical yearbooks, and China Energy Statistical Yearbook. This paper uses Stata 16 software for analysis.

### 3.2. Variable Setting

#### 3.2.1. Interpreted Variable

Carbon emissions (*C*). Among the greenhouse gases (GHGs) that cause climate change, carbon dioxide content accounts for the highest proportion, and the greenhouse effect is the most significant [22]. Other greenhouse gases (such as methane) can also be expressed in the form of carbon dioxide.

Therefore, this paper uses carbon dioxide emissions to measure regional carbon emissions. This paper calculates the carbon emission level ($C_{it}$) of Chinese provinces according to the IPCC Guidelines for National Greenhouse Gas Inventories in 2006 and the IPCC Guidelines for National Greenhouse Gas Inventories in the 2019 revision prepared by the Intergovernmental Panel on Climate Change (IPCC) [23,24]. The calculation formula is:

(1) $$C_{it}=\sum_{i=1}^{n}M_{i}\times p_{i}\times q_{i}\times o_{i}\times \frac{44}{12}$$

In the above equation, $C_{it}$ represents the total carbon emissions of each province. $M_{i}$ is the consumption of the *i* energy in each province. $P_{i}$ is the average low calorific value of the *i* energy. $q_{i}$ is the carbon content of the *i* energy unit calorific value. $O_{i}$ is the carbon oxidation rate of the *i* energy.

#### 3.2.2. Core Explanatory Variables

Financial agglomeration (*fa*). This paper selects the location entropy index to measure the financial agglomeration level of 30 provinces (autonomous regions, municipalities directly under the Central Government) in China.

(2) $$fa_{it}=\frac{e_{it}/p_{it}}{E_{t}/P_{t}}$$

where *e_it_* is the number of financial industry employees in region *i*. *P_it_* is the total number of employees in region *i* during period *t*. *E_t_* refers to the number of financial industry employees in the period *t*. Pt refers to the total number of employees at the time of period *t*. If the financial location entropy is greater than 1, it indicates that the financial industry in this region has a comparative advantage over the whole country. If the location entropy is less than 1, it indicates that the local financial industry does not have the advantage of professional development compared with the whole country.

#### 3.2.3. Mediated Variable

This paper selects two indicators of the proportion of secondary and third industries as intermediary transmission variables. We measure the proportion of secondary industry (*ind*) by dividing the output value of the regional secondary industry by the GDP of the region. We measure the proportion of third industries (*third*) by dividing the output value of the regional third industries by the GDP of the region.

#### 3.2.4. Control Variables

We chose the population size (*lnp*), the degree of opening up (*lndow*), the level of economic development (*lngdp*), the degree of government intervention (*gov*), and the degree of education (*edu*) as control variables. We take the logarithm of the number of permanent residents as a measure of the regional population size. This paper uses the proportion of the total import and export value in the total local product and takes logarithms to measure the degree of regional opening to the outside world. We measure the level of regional development by taking the logarithm of GDP. This paper uses the proportion of government fiscal expenditure to GDP to measure the degree of government intervention. We use the average years of education to measure the level of education in the region. Table 1 shows the results of descriptive statistics of variables.

### 3.3. Model Setting and Empirical Strategies

#### 3.3.1. Exploratory Spatial Data Analysis

Exploratory Spatial Data Analysis (ESDA) uses spatial data analysis techniques and methods to visually describe the spatial distribution rules and mechanisms of the research objects, mainly including the global Moran’s I index, local Moran’s I, and LISA aggregation map. ESDA can accurately measure the spatial distribution and mechanisms of China’s total carbon emissions.

The global Moran’s I index mainly explores the spatial distribution characteristics of research objects in the whole region. The index ranges from −1 to 1. The strength of the spatial connection increases as the absolute value increases. Moran’s I > 0 indicates a positive spatial correlation; otherwise, it indicates a negative correlation, and being close to 0 means no spatial autocorrelation. The calculation formula is as follows:

(3) $$I=\frac{N\sum_{i}\sum_{j}W_{ij}\left(X_{i}-\bar{X}\right)\left(X_{j}-\bar{X}\right)\;}{(\sum_{i}\sum_{j}W_{ij})\sum_{i}{\left(X_{i}-\bar{X}\right)}^{2}}$$

Local Moran’s I mainly explore the spatial heterogeneity of China’s total carbon emissions in sub-regions and measure the degree of correlation between region and its neighbors. In the formula, the accumulation of *j* does not include the region *i* itself, that is, *j* ≠ *i*. The calculation formula is as follows:

(4) $$\begin{array}{l} I_{i}=\frac{\left(X_{i}-\bar{X}\right)}{S_{x}{}^{2}}\sum_{j}\left[W_{ij}\left(X_{j}-\bar{X}\right)\;\right]\;\\ \;S_{x}^{2}=\sum_{j}\left(X_{j}-\bar{X}\right)\;/N \end{array}$$

In the above formula, *N* is the total number of provinces under study; $X_{i}$ and $X_{j}$ are the total carbon emissions of regions  i and j; $W_{ij}$ is the spatial weight matrix; $\bar{X}$ is the average of total carbon emissions.

The LISA cluster graph combines Moran scatter plot and local Moran’s I index to graphically visualize the cluster type and significance level of the research object.

#### 3.3.2. Datum Model

This paper uses a panel model to measure the direct impact of financial agglomeration on carbon emissions. Therefore, our model was built as follows:

(5) $$C_{it}=\alpha_{0}+\alpha_{1}\;fa_{it}+\sum \alpha X_{it}+u_{i}+\mu_{i}+\varepsilon_{i,t}$$

where *i* and *t* in the equations above represent provinces and years, respectively. $C_{it}$ represents the total carbon dioxide emissions. fa indicates the degree of regional financial agglomeration. $X_{it}\;$ represents the control variable. $\alpha_{0}$ is a constant item. $u_{i}$ is the fixed effect of the province. $\mu_{t}$ is the fixed effect of the year. $\varepsilon_{it}$ is the residual item. The benchmark model controls both time and provincial fixed effects and adopts the robust standard error clustered to the provincial level.

#### 3.3.3. Mediation Effect Model

The above model analyzes the direct impact of financial agglomeration on regional carbon emissions. However, further research is still needed to determine the precise process by which financial agglomeration affects carbon emission levels. Therefore, this paper chooses the percentage of secondary and third industries as its two channels for verification. In this paper, recursive equations (Equations (6) and (7)) are set to identify the specific impact mechanism of financial agglomeration on regional carbon emissions.

(6) $$M_{it}=\beta_{0}+\beta_{1}\;fa_{it}+\beta_{2}X_{it}+u_{i}+\mu_{t}+\varepsilon_{it}$$

(7) $$C_{it}=\lambda_{0}+\lambda_{1}\;fa_{it}+\lambda_{2}M_{it}+\lambda_{3}X_{it}+u_{i}+\mu_{t}+\varepsilon_{it}$$

In the above formula, M is the intermediary variable, which is respectively the proportion of secondary industry and third industry. The definitions of other variables are the same as those in Equation (5). $\beta_{1}\times \lambda_{2}$ represents the intermediary effect, indicating the impact of financial agglomeration on regional carbon emissions through the intermediary variables.

#### 3.3.4. Adjustment Effect Test

Europe, the United States, and other countries have actively tried to use the carbon emission trading market mechanism to solve the problem of climate change. The government sets the overall quota for the carbon trading market’s “carbon emission rights”, which are the market’s subject matter. The government will assign tradable carbon emission quotas to emission control organizations after the total has been calculated. On the trading market, the quota is available for purchase and sale. Enterprises with high carbon emissions will buy carbon emission quotas from the market. Therefore, carbon emission trading increases the production cost of high-carbon enterprises, forcing them to reduce carbon emissions. China is also actively trying to establish a carbon emission trading market to govern China’s carbon emission level. The main topic to be addressed in this section is whether financial agglomeration can demonstrate an effective emission reduction effect under the pilot policy of carbon emission trading. To answer this question, we designed the following model for testing:

(8) $$C_{it}=\beta_{0}+\beta_{1}\;fa_{it}+\beta_{2}did+\sum \beta {\;X}_{it}+u_{i}+\mu_{t}+\varepsilon_{it}$$

(9) $$C_{it}=\lambda_{0}+\lambda_{1}\;fa_{it}+\lambda_{2}did+\lambda_{3}fa\times did+\sum \lambda X_{it}+u_{i}+\mu_{t}+\varepsilon_{it}$$

where did represents the policy variable. If the province becomes a pilot area in this year, the province’s did in this year is set to 1; otherwise, it is set to 0. Because the signing of the official documents of the carbon trading pilot project has a “predictive” effect on the public’s policy direction, this paper divides the starting time of the carbon trading mechanism based on the signing of official documents of the carbon trading pilot. The definitions of other variables are the same as those in Equation (5).

#### 3.3.5. Spatial Effect Model

This paper uses the spatial Dubin model to analyze the impact of financial agglomeration on carbon emissions under the spatial effect. Our model was built as follows:

(10) $$C_{it}=\alpha_{0}+\rho WC_{it}+\alpha_{1}fa_{it}+\varphi_{1}Wfa_{it}+\sum \alpha X_{it}+\sum \varphi WX_{it}+u_{i}+\mu_{t}+\varepsilon_{it}$$

where ρ and *W* represent the spatial autocorrelation coefficient and the spatial weight matrix. $\varphi_{1}$ is the coefficient of the spatial interaction term of the core explanatory variable. *φ* is the coefficient of the spatial interaction term of control variables. Other variable settings are shown in Formula (5). We use the adjacency matrix as the spatial weight matrix to verify the spatial effect of financial agglomeration on carbon emissions. At the same time, we use a geographic distance matrix and economic geography matrix to test the robustness.

## 4. Imperial Results and Analysis

### 4.1. Spatial and Temporal Distribution

Figure 1 shows the changing trend of total carbon emissions and carbon emission intensity. That means China’s total carbon emissions are rising, while the carbon emission intensity is declining. However, it cannot reveal the spatial distribution characteristics of China’s carbon emissions. Therefore, this paper uses ESDA for analysis.

**Figure 3:**
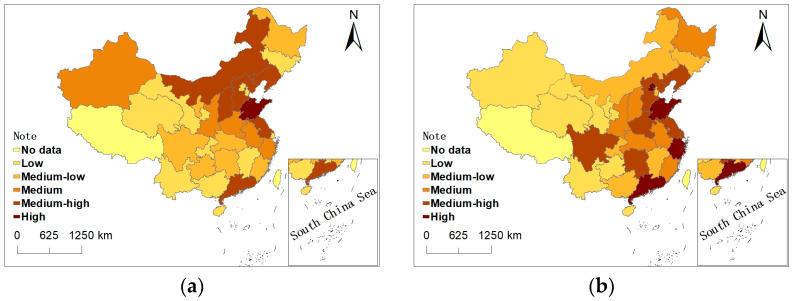
(**a**) Spatial distribution of total carbon emissions in 2018; (**b**) spatial distribution of financial employees in 2018.

Figure 3a shows that China’s regions with high carbon emissions are concentrated in Inner Mongolia, Shanxi, Hebei, Liaoning, Shandong, Jiangsu, and Guangdong. The provinces with low carbon emissions are concentrated in Qinghai, Yunnan, Gansu, Chongqing, Jiangxi, Guangxi, Hainan, Beijing, Tianjin, and Shanghai. Figure 3b shows that the provinces with a large number of financial practitioners mainly include Beijing, Shandong, Zhejiang, Shanghai, Guangdong, Hunan, Henan, Hebei, Liaoning, Jiangsu, and Sichuan. The provinces with a small number of financial practitioners are mainly concentrated in Xinjiang, Qinghai, Gansu, Ningxia, Yunnan, and Guizhou.

Figure 3 means that China’s carbon emissions and finances have significant spatial distribution characteristics. We use the LISA chart to analyze the concentration level of China’s carbon emissions and financial practitioners. This paper concludes with the following four types: The first type is the H–H cluster type, indicating that the variables of the province are high and that the surrounding provinces are also high, showing a positive correlation. The second type is the H–L agglomeration type, indicating that the variables of the province are high and that the surrounding provinces are low, showing a negative correlation. The third type is the L–H air core type, indicating that the variables of the province are low and that the surrounding provinces are high. The fourth type is L–L agglomeration, indicating that the variables of the province are low and that the surrounding provinces are low.

**Figure 4:**
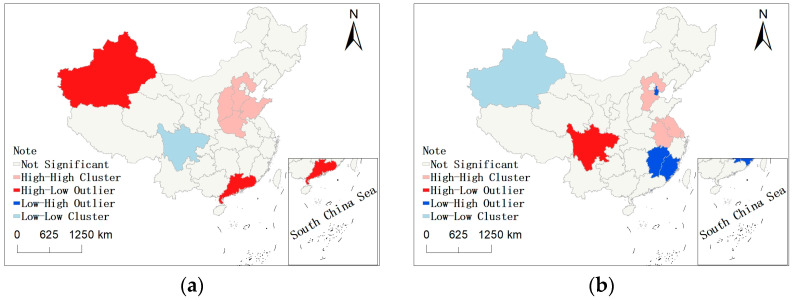
(**a**) LISA chart of total carbon emissions in 2018; (**b**) LISA chart of financial employment in 2018.

Figure 4a shows the LISA chart of total carbon emissions in 2018. The results show that the H–H concentration areas mainly include Shandong, Hebei, Shanxi, and Henan. The L–L cluster area is distributed in Sichuan. The H–L concentration areas mainly include Guangdong. Figure 4b shows the LISA chart of financial employment in 2018. The results show that the H–H concentration areas mainly include Jiangsu, Anhui, and Hebei. The L–L cluster area is distributed in Xinjiang. The H–L concentration areas mainly include Sichuan. The L–H cluster area is concentrated in Fujian, Jiangxi, and Tianjin.

### 4.2. Benchmark Regression Results

In Table 2, when we do not add control variables, the coefficient of financial agglomeration is −8.972, which is significant at a significance level of 1%. Model (4) shows that the coefficient of the financial agglomeration is −7.855, which is significant at a significance level of 5%. Models (1)–(4) shows that financial agglomeration helps to reduce regional carbon emissions. We believe that the emission-reduction effect of financial agglomeration is significant. The reasonable spatial layout of the financial industry is conducive to carbon peaking and carbon neutralization. This paper believes that financial agglomeration can promote the development of low-energy consumption industries and green industries in the region, improve energy consumption efficiency, and reduce the level of carbon emissions in the region. Secondly, financial agglomeration can promote the development of a low-carbon circular economy, promote the establishment of a carbon emission trading fund, and give play to the financial attributes of the carbon market. In addition, policies such as regulating high-carbon enterprises and credit support for environmental protection enterprises promote the transfer of financial resources to low-carbon emission enterprises. The research conclusion confirms hypothesis H0.

### 4.3. Robustness Test

To ensure the accuracy of the results, this paper conducts a robustness test. In Table 3, model (1) is the regression results of replacing the explained variable with per capita carbon emissions. The model (1) results show that the coefficient of financial agglomeration is −3.835, which is significant at the 1% significance level. That means financial agglomeration can reduce carbon emissions per capita. Model (2) is the regression results of the explained variables replaced by the logarithm of the total carbon emissions. The model (2) results show that the coefficient of financial agglomeration is −0.181, which is significant at the 1% significance level. That means that financial agglomeration can reduce the growth rate of carbon emissions.

To reduce the errors caused by missing variables, in model (3), the control variables increase the provincial innovation level (rd), post and telecommunications business (youdian), and foreign direct investment (waistz). The model (3) results show that the coefficient of financial agglomeration is −7.085, which is significant at the 1% significance level. Next, we use the lag term of financial agglomeration as an explanatory variable to consider the reverse causality. The results of model (4) show that the coefficient of the lag term of financial agglomeration is −8.569, which is significant at the 1% level.

### 4.4. Heterogeneity Test

We divided China into three regions: the east, the middle, and the west, to verify the heterogeneous impact of financial agglomeration on carbon emissions. According to model (1) in Table 4, the financial agglomeration coefficient in the eastern provinces is −0.140, which is not statistically significant. Model (2) shows that the coefficient of financial agglomeration is −12.968, which is significant at the 5% significance level in central provinces. Model (3) reveals that the coefficient of financial agglomeration is −8.053, which is not significant in western provinces. Models (1), (2), and (3) show that financial agglomeration has an emission reduction effect only on central provinces. Although green technology, financial concentration, and economic development are all strong in eastern China, the total carbon emissions are still high because of population concentration, excessive energy consumption, and industrial concentration. Therefore, the emission reduction effect of financial agglomeration in eastern China is insignificant. The financial system in central China is developed and relatively stable, which can promote green technology innovation, improve energy efficiency, and improve the low-carbon economy. As a result, financial agglomeration can significantly reduce emissions in central China. This paper argues that the financial system in the western region is not active enough, and green funds cannot accurately move to low-carbon enterprises. At the same time, the western region is short on talent and technology, so it is difficult to realize the transformation of low-carbon technology. Therefore, the emission reduction effect of financial agglomeration in western China is not significant.

We regard a region as high-carbon if its total carbon emissions are higher than the average for this year. Otherwise, it will be regarded as a low-carbon region. Model (4) in Table 4 shows that the coefficient of financial agglomeration is 6.288, which is not significant in a high-carbon region. Model (5) shows that the coefficient of financial agglomeration is −2.529, which is significant at the level of 1% significance. Financial agglomeration dramatically reduces emissions in low-carbon countries by providing funds for green technology innovation and improved energy efficiency. Meanwhile, the transformation of green industrial structures in high-carbon regions is slow, and their economic growth is highly dependent on resources, with an obvious carbon-locking effect. Therefore, the emission reduction effect of financial agglomeration in high-carbon areas is not significant.

### 4.5. Mechanism Identification Test

We select the proportion of secondary and third industries to test the impact mechanism of the financial agglomeration on carbon emissions. In the process of verification, we controlled the year-fixed effect. Model (1) in Table 5 shows that the coefficients of financial agglomeration are negative and significant at the 1% significance level. That means that the financial agglomeration reduces the proportion of secondary industries. Model (2) shows that the coefficient of the proportion of secondary industry is 66.229, which is significant at the level of 1% significance. That shows that the proportion of secondary industry has increased carbon emissions. The results of models (1) and (2) mean that the financial agglomeration can inhibit regional carbon emissions by reducing the proportion of secondary industry. Financial agglomeration can promote enterprise development, intensify enterprise competition, and enhance the preference for environment-friendly industries. That led to the transfer of the secondary industry with high carbon emissions to other regions. Therefore, financial agglomeration reduces carbon emissions by reducing the secondary industry. The research conclusion confirms hypothesis H1.

Model (3) shows that the coefficient of financial agglomeration is 0.073 and is highly significant. It shows that financial agglomeration promotes the proportion of third industries. Model (4) shows that the coefficient of the proportion of third industries is −65.203, which is significant at a significance level of 1%. That means that the proportion of third industries can inhibit regional carbon emissions. The results of models (3) and (4) mean that the financial agglomeration can inhibit regional carbon emissions by promoting the proportion of third industries. We believe that financial agglomeration has promoted the development of the third industry by reducing the secondary industry. The third industry has a high added value and low emissions. Finance can provide funds for developing the third industry, resulting in economies of scale. Therefore, financial agglomeration can reduce carbon emissions by increasing the proportion of the third industry. The research conclusion confirms hypothesis H2.

### 4.6. Emission Reduction Effect of Financial Agglomeration under Carbon Emission Trading

In Table 6, model (1), the coefficients of fa and did were −7.142 and −9.237, respectively, and both were significant at a 1% significance level. In model (2), the coefficient of fadid is −2.785, which is significant at a 1% significance level. The results show that the pilot carbon emission trading policy has strengthened the negative impact of financial agglomeration on carbon emissions. According to this study, carbon-emission rights can act as a financial tool to optimize resource allocation, modify the economic system, and encourage the transition of sectors from high- to low-carbon supply chains. Consequently, carbon emission trading can enhance the financial agglomeration’s ability to reduce emissions. The findings of the study support hypothesis H3.

### 4.7. Spatial Effect Test

This paper uses the spatial Dubin model to test the spatial spillover effect of financial agglomeration on carbon emissions. First, we conduct spatial autocorrelation analysis based on Moran’s I index. The results of the Moran’s I index are shown in Table 7. The Moran’s I indices under the adjacency matrix are all greater than 0 and are generally significant at a 5% significance level. That shows that regional carbon emissions have a positive spatial correlation, and it is necessary to conduct spatial analysis in carbon emissions research.

Second, we use the LM test to select the SEM or SAR. Table 8 shows that we should use the SEM. Elhorst (2010) pointed out that, if the LM test results support either or both the SEM and the SAR, we should use the SDM [25]. Then, this paper conducts an LR test to determine whether the spatial Dubin model will degenerate into an SEM or a SAR. The LR test results show that the SDM cannot be simplified into an SEM or SAR. Therefore, we use the SDM to test the spatial effect of financial agglomeration on regional carbon emissions. The Wald test was carried out in this paper. The test result shows that Prob>chi2 = 0.0000. That means that the spatial measurement model should be set as the SDM. Finally, the results of the Hausman test show that we should select the SDM with a bidirectional fixed effect.

Table 9 shows the regression results of utilizing an adjacency matrix, a geographic distance matrix, and an economic geography matrix. The coefficients of financial agglomeration in models (1)–(3) are −8.071, −13.511, and −7.353, respectively, and are significant at the 1% significance level. That means that financial agglomeration can still restrain regional carbon emissions after considering the spatial effect. The coefficients of population growth rate are respectively −23.770, −50.816, and −37.352, which are significant at least at the 5% significance level. This paper believes that population growth is suitable for economic development and environmental pollution control. Therefore, appropriate population growth will reduce carbon emissions. Meanwhile, the coefficients of government intervention degree are respectively −23.770, −50.816, and −37.352, which are significant at a 1% significance level. This paper argues that, the greater the government’s ability to intervene, the greater the impact of government emission reductions. Because the government can impose stricter emission reduction measures on high-carbon firms and improve energy structure transformation and upgrading. It is worth noting that, in model (3), the coefficient of Wfa is 25.036, which is significant at a 1% significance level. We believe that financial agglomeration makes enterprises with high carbon emissions move to regions with similar economic geography. That increases carbon emissions in regions with similar economic geography.

## 5. Conclusions and Suggestions

### 5.1. Conclusions

Controlling carbon emissions has become a global consensus. However, the existing literature rarely explores the impact of financial agglomeration on carbon emissions. Based on the analysis of the emission reduction history of major countries, this paper mainly uses the provincial-level data of China from 2002 to 2018 to study the impact, impact mechanisms, and spatial effects of financial agglomeration on regional carbon emissions. The following are the conclusions:

First, this paper analyzes the situation of carbon emissions and emission reduction policies in China and major developed countries. China lacks carbon tax policies, and there are many drawbacks in the carbon trading market; a “bottom-up” voluntary emission reduction mechanism has not been formed. Compared with other developed countries, China has a low per capita income, a low urbanization rate, a high proportion of secondary industry, and high coal consumption.

Second, we conduct research using ESDA and discover that the majority of China’s carbon-emitting provinces are located in Inner Mongolia, Shanxi, Hebei, Liaoning, Shandong, Jiangsu, and Guangdong. The region with huge financial practitioners includes Beijing, Shandong, Zhejiang, Shanghai, Guangdong, Hunan, Henan, Hebei, Liaoning, Jiangsu, and Sichuan. The total carbon emissions and financial practitioners have characteristics of spatial agglomeration.

Third, our empirical study found that financial agglomeration can reduce carbon emissions and passed a series of robustness tests. The heterogeneity analysis shows that the emission reduction effect of financial agglomeration is more significant in the central and high-carbon regions. The intermediary mechanism test found that financial agglomeration can reduce carbon emissions by reducing the secondary industry’s proportion and increasing the third industry’s proportion. The adjustment mechanism found that the emission reduction effect of financial agglomeration under carbon trading is more significant. It is worth noting that financial agglomeration can still reduce carbon emissions after considering the spatial effect. We find that financial agglomeration in regions with close economic geography will increase the region’s total carbon emissions.

### 5.2. Suggestions

Based on the conclusions of this paper, we propose the following suggestions.

We first propose that China develop a sensible carbon tax policy, enhance the carbon trading mechanism, recruit qualified carbon trading professionals, work toward establishing a “bottom-up” voluntary emission reduction mechanism, assume the role of financial resource allocation, and direct funds toward environmentally friendly industries.

Second, China needs to place severe limits on carbon production in high-carbon areas. To that end, we urge low-carbon areas to share their knowledge on emissions reduction with high-carbon areas. It is important to remember that high-carbon businesses should not be allowed to relocate to low-carbon regions. Meanwhile, we advocate for standardizing the spatial layout of industrial structure.

Third, financial agglomeration can reduce the proportion of the secondary industry’s proportion and increase the third industry’s proportion. Consequently, we need to encourage the green transformation and upgrading of the secondary industry and reduce capital credit to industries with high pollution, high energy consumption, and high emissions. Financial institutions should provide green credit for low-carbon environmental protection enterprises and industries.

Fourth, carbon emission trading plays an important role in the process of financial agglomeration to curb regional carbon emissions. Therefore, we should encourage carbon emissions trading, urge financial institutions to provide carbon financial derivatives transactions, establish a unified large carbon trading market in China, and promote market-oriented carbon trading.

Fifth, financial institutions should vigorously promote green credit, green financial products, and mechanism innovation. We encourage financial institutions to give play to the financial attributes of the carbon trading market, promote the innovation of carbon financial products, improve the liquidity of the carbon trading market, enrich the product portfolio, and provide hedging tools.

The main limitations of this study are: cities account for 2% of the earth’s surface, but their residents consume 75% of the world’s energy resources [26]. Reducing emissions in cities is crucial. However, due to data availability, this paper mainly analyzes the data at the provincial level in China. In the future, we will further explore the emission reduction mechanism at the urban level.

## Figures and Tables

**Table 1:** Descriptive statistics.

Variable	Obs	Mean	Std. Dev.	Min	Max
C	510	33.858	25.946	0.606	149.307
fa	510	1.212	0.851	0.420	5.449
ind	510	0.460	0.081	0.186	0.615
third	510	0.424	0.088	0.286	0.810
lnp	510	8.166	0.752	6.270	9.337
lndow	510	5.552	0.994	3.211	7.842
lngdp	510	9.156	1.086	5.797	11.485
gov	510	0.210	0.094	0.079	0.627
edu	510	8.642	1.032	6.041	12.555

**Table 2:** Benchmark regression.

Variables	(1)	(2)	(3)	(4)
	C	C	C	C
fa	−8.972 ***	−6.696 *	−8.001 **	−7.855 **
	(−3.248)	(−1.970)	(−2.205)	(−2.198)
lnp		−22.515	−28.875	−28.680
		(−1.043)	(−1.199)	(−1.187)
lndow		−4.822	−5.515 *	−5.544 *
		(−1.299)	(−1.882)	(−1.881)
lngdp		−6.175	−9.688	−9.279
		(−0.607)	(−1.002)	(−1.032)
gov			−98.010 **	−98.059 **
			(−2.532)	(−2.538)
edu				−0.997
				(−0.226)
Constant	44.736 ***	309.154	419.232 *	422.511 *
	(13.358)	(1.499)	(1.803)	(1.785)
Year	Yes	Yes	Yes	Yes
Province	Yes	Yes	Yes	Yes
Observations	510	510	510	510
R-squared	0.8989	0.9011	0.9115	0.9114

T statistics in parentheses; *** *p* < 0.01, ** *p* < 0.05, * *p* < 0.1.

**Table 3:** Robustness test.

Variables	(1)	(2)	(3)	(4)
	PC	lnC	C	C
fa	−3.835 ***	−0.181 ***	−7.085 ***	
	(−6.108)	(−2.774)	(−4.558)	
layfa				−8.569 ***
				(−5.502)
lnp	−1.093	−0.154	−27.176 ***	−24.789 ***
	(−0.352)	(−0.718)	(−3.481)	(−2.795)
lndow	−2.402 ***	−0.061 *	−5.153 ***	−4.992 ***
	(−4.681)	(−1.805)	(−3.748)	(−3.748)
lngdp	7.007 ***	0.644 ***	−5.702	−10.816 **
	(3.954)	(4.939)	(−1.388)	(−2.578)
gov	5.535	0.915 **	−65.916 ***	−87.218 ***
	(1.172)	(2.128)	(−5.019)	(−5.929)
edu	−0.299	−0.081 *	−3.921 *	1.306
	(−0.430)	(−1.900)	(−1.781)	(0.635)
rd			−0.250 *	
			(−1.957)	
youdian			0.865	
			(0.698)	
waistz			0.025 ***	
			(4.938)	
Constant	−27.077	−0.338	405.103 ***	382.761 ***
	(−0.871)	(−0.151)	(4.820)	(4.329)
Year	Yes	Yes	Yes	Yes
Province	Yes	Yes	Yes	Yes
Observations	510	510	480	480
R-squared	0.8613	0.9586	0.9309	0.9250

T statistics in parentheses; *** *p* < 0.01, ** *p* < 0.05, * *p* < 0.1.

**Table 4:** Heterogeneity test.

Variables	(1)	(2)	(3)	(4)	(5)
	C	C	C	C	C
fa	−0.140	−12.968 **	−8.053	6.288	−2.529 ***
	(−0.063)	(−2.313)	(−1.293)	(1.450)	(−3.914)
lnp	−96.043 ***	52.191	37.721 ***	−67.086 **	−17.117 ***
	(−6.591)	(1.417)	(2.907)	(−2.383)	(−5.169)
lndow	−7.937 **	−7.021 ***	−1.652	−14.548 ***	−0.055
	(−2.082)	(−2.678)	(−0.961)	(−4.267)	(−0.096)
lngdp	−3.122	−3.880	−6.445	8.788	4.055 **
	(−0.406)	(−0.543)	(−1.062)	(0.985)	(2.282)
gov	−182.856 ***	−92.229 *	−61.943 ***	11.786	−27.019 ***
	(−4.517)	(−1.695)	(−4.237)	(0.203)	(−5.084)
edu	−7.775	11.856 ***	−9.362 ***	−5.195	−0.366
	(−1.411)	(3.159)	(−3.634)	(−0.975)	(−0.462)
Constant	1014.890 ***	−404.141	−110.414	672.778 **	132.468 ***
	(6.904)	(−1.320)	(−1.051)	(2.160)	(4.149)
Year	Yes	Yes	Yes	Yes	Yes
Province	Yes	Yes	Yes	Yes	Yes
Observations	204	153	153	179	329
R-squared	0.9266	0.9068	0.8486	0.8987	0.9337

T statistics in parentheses; *** *p* < 0.01, ** *p* < 0.05, * *p* < 0.1.

**Table 5:** Inspection of intermediary mechanism.

Variables	(1)	(2)	(3)	(4)
	ind	C	third	C
fa	−0.044 ***		0.073 ***	
	(−5.554)		(13.149)	
ind		66.229 ***		
		(5.039)		
third				−65.203 ***
				(−5.115)
lnp	−0.163 ***	−2.155	0.060 ***	−9.417 ***
	(−14.802)	(−0.468)	(7.595)	(−2.608)
lndow	−0.042 ***	−6.732 ***	0.032 ***	−7.129 ***
	(−8.337)	(−4.719)	(11.265)	(−5.136)
lngdp	0.186 ***	17.897 ***	−0.062 ***	26.596 ***
	(12.974)	(3.580)	(−7.467)	(7.093)
gov	0.083	−26.965 *	0.042	−12.006
	(1.392)	(−1.726)	(0.890)	(−0.773)
edu	−0.025 ***	1.539	0.003	1.462
	(−3.762)	(0.779)	(0.552)	(0.780)
Constant	0.567 ***	−113.135 ***	0.200 ***	−75.679 ***
	(6.492)	(−4.339)	(3.023)	(−3.626)
Observations	510	510	510	510
R-squared	0.5196	0.5052	0.7799	0.4989

T statistics in parentheses; *** *p* < 0.01, * *p* < 0.1.

**Table 6:** Test results of regulation mechanism.

Variables	(1)	(2)
	C	C
fa	−7.142 ***	−4.099 *
	(−4.760)	(−1.824)
did	−9.237 ***	−4.958 ***
	(−6.353)	(−2.879)
fadid		−2.785 ***
		(−2.631)
lnp	−6.590	0.688
	(−0.757)	(0.070)
lndow	−4.721 ***	−5.145 ***
	(−3.619)	(−3.942)
lngdp	−8.687 **	−10.666 ***
	(−2.245)	(−2.589)
gov	−106.932 ***	−106.280 ***
	(−7.307)	(−7.290)
edu	−0.709	−1.215
	(−0.296)	(−0.504)
Constant	231.549 ***	193.305 **
	(2.694)	(2.138)
Observations	510	510
R-squared	0.9156	0.9163

T statistics in parentheses; *** *p* < 0.01, ** *p* < 0.05, * *p* < 0.1.

**Table 7:** The Moran’s I index.

Year	I	E(I)	Sd(I)	Z	*p*-Value
2002	0.203	−0.035	0.122	1.945	0.052
2003	0.187	−0.035	0.122	1.819	0.069
2004	0.225	−0.035	0.123	2.119	0.034
2005	0.263	−0.035	0.122	2.447	0.014
2006	0.255	−0.035	0.122	2.383	0.017
2007	0.258	−0.035	0.121	2.411	0.016
2008	0.266	−0.035	0.121	2.494	0.013
2009	0.243	−0.035	0.121	2.299	0.022
2010	0.244	−0.035	0.120	2.313	0.021
2011	0.243	−0.035	0.122	2.273	0.023
2012	0.234	−0.035	0.121	2.224	0.026
2013	0.223	−0.035	0.121	2.123	0.034
2014	0.214	−0.035	0.120	2.064	0.039
2015	0.224	−0.035	0.119	2.177	0.030
2016	0.210	−0.035	0.118	2.076	0.038
2017	0.196	−0.035	0.118	1.955	0.051
2018	0.198	−0.035	0.119	1.954	0.051

**Table 8:** Spatial effect test.

Test	Statistic	df	*p*-Value
Spatial error:			
Moran’s I	2.752	1	0.006
Lagrange multiplier	120.186	1	0.000
Robust Lagrange multiplier	63.207	1	0.000
Spatial lag:			
Lagrange multiplier	57.021	1	0.000
Robust Lagrange multiplier	0.042	1	0.838

**Table 9:** Regression results of spatial econometric model.

Variables	(1)	(2)	(3)
	C	C	C
fa	−8.071 ***	−13.511 ***	−7.353 ***
	(−4.55)	(−7.19)	(−4.23)
lnp	−23.770 **	−50.816 ***	−37.352 ***
	(−2.25)	(−5.81)	(−4.34)
lndow	−2.613 *	−1.876	−6.363 ***
	(−1.81)	(−1.28)	(−4.41)
lngdp	−4.421	−9.396 **	−10.215 ***
	(−1.16)	(−2.54)	(−2.59)
gov	−131.632 ***	−80.420 ***	−115.579 ***
	(−9.15)	(−5.91)	(−7.57)
edu	−1.226	0.511	−1.925
	(−2.35)	(1.77)	(0.41)
Wfa	−3.169	−17.685	25.036 ***
	(−0.81)	(−1.54)	(6.24)
Wlnp	8.214	553.057 ***	−68.634 ***
	(0.42)	(9.08)	(−3.38)
Wlndow	3.583	15.971	−11.210 ***
	(1.31)	(1.58)	(−3.21)
Wlngdp	−14.100 **	5.556	39.836 ***
	(−2.25)	(0.27)	(4.48)
Wgov	143.346 ***	253.058 ***	−66.417 ***
	(6.06)	(3.18)	(−2.66)
Wedu	−9.347 **	24.794 *	1.970
	(−2.35)	(1.77)	(0.41)
rho	0.283 ***	−0.104	0.283 ***
	(4.52)	(−0.58)	(3.78)
sigma2_e	47.080 ***	44.159 ***	45.596 ***
	(15.69)	(15.96)	(15.73)
Observations	510	510	510
R-squared	0.264	0.015	0.083

T statistics in parentheses; *** *p* < 0.01, ** *p* < 0.05, * *p* < 0.1.

## Data Availability

The raw data supporting the conclusion of this article will be made available by the authors without undue reservation.
